# Implementation of artificial intelligence in the 2025 medical parasitology course at Hallym University

**DOI:** 10.3352/jeehp.2026.23.4

**Published:** 2026-02-05

**Authors:** Eun Hee Ha

**Affiliations:** College of Medicine, Hallym University, Chuncheon, Korea; The Catholic University of Korea, Korea

As artificial intelligence (AI) continues to develop, academics are increasingly integrating AI into education to increase efficiency and promote personalized learning [1]. ChatGPT (https://chatgpt.com/) has demonstrated substantial potential to transform medical education [2]. As the role of AI in medicine expands, medical education must likewise evolve to prepare future physicians to adapt to societal and technological changes [3]. This correspondence describes my experience as a third-year medical student participating in an AI-Implemented Medical Parasitology (AIMP) course from October 27 to December 15, 2025, at Hallym University in South Korea.

## AI-Implemented Medical Parasitology course curriculum

**Fig. 1.**
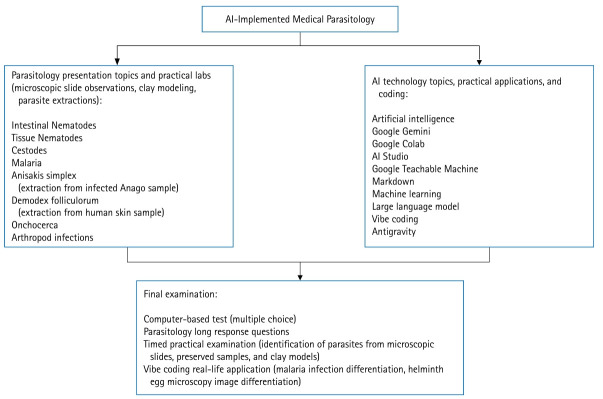
Flowchart of the artificial intelligence (AI)-Implemented Medical Parasitology (AIMP) course curriculum integrating parasitology and AI technology (generated using Flow.io by the author). Each week, students lead a 2-hour learning session, conduct a 2-hour laboratory experiment, and participate in a 2-hour AI laboratory, totaling 6 hours per week over a 7-week period. The course is jointly led by 2 professors: a parasitologist and an information technology specialist.

The AIMP course curriculum differed markedly from that of conventional medical school courses (Fig. 1, Supplement 1). Its most distinctive feature was the emphasis on introducing students to emerging AI technologies and encouraging their use to foster independent learning in parasitology. The course adopted a flipped-learning approach and was uniquely structured, with professors largely entrusting students to acquire new course material through group discussions and presentations. In many sessions, only minimal prompts were provided, and students were encouraged to collaboratively discuss, research, and present prompt-related information to the class. Each week focused on a different parasitology topic, followed by laboratory experiments ranging from microscopic observations to parasite extraction from provided materials. In addition, a dedicated AI technology component was taught weekly to introduce new AI tools and reinforce foundational knowledge in both AI and parasitology. Although this approach was initially unfamiliar and challenging, the integration of wet-lab practical work with AI-based analyses promoted active learning and critical thinking. Compared with passive lecture-based note-taking or purely wet-lab practicals in traditional courses, the inclusion of AI motivated deeper engagement with the course content and encouraged a more multifaceted approach to parasitology.

## Introduction of AI tools

The AI tools introduced during the course were particularly beneficial, as they facilitated alternative approaches to understanding concepts and retaining knowledge. For example, NotebookLM (https://notebooklm.google.com/), an AI research tool developed by Google, generated summaries, flashcards, and mini-quizzes from course materials. Gemini (https://gemini.google.com/), another Google AI tool designed for deep searching, supported research by producing structured responses while clearly indicating the reference sources used. AI Studio (https://aistudio.google.com/), a more advanced platform that implements Gemini, enabled finer adjustments to system settings and allowed the use of custom instructions tailored to specific user needs. Owing to their practical functionality, Gemini and AI Studio were widely used for parasitology-related research within the AIMP course. The summarized outputs generated by these tools allowed me to concentrate on understanding core concepts rather than navigating large volumes of course-irrelevant material from published articles. Overall, AIMP equipped me with AI tools that can be applied across multiple stages of the learning process.

AIMP also introduced me to the practical applications of AI and its potential impact on the future of parasitology research. When I first encountered Teachable Machine (https://teachablemachine.withgoogle.com/), I was eager to explore its capabilities. This tool enabled the development of AI models that distinguish between 2 classifications using visual inputs, which is particularly advantageous for research involving the differentiation of microscopic slide images. Beginning with a simple task of building a model to classify cats and dogs, I learned the appropriate use of the tool and subsequently applied those skills to more complex, real-world research tasks.

## Final exam: deep learning case study of public data

**Fig. 2.**
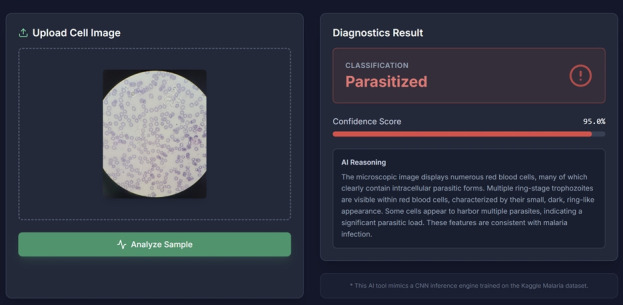
Vibe coding–based artificial intelligence (AI) model generated in AI Studio for differentiating malaria parasitic infection. The uploaded cell image was obtained from a *Plasmodium vivax* microscopic specimen collected during the AI-Implemented Medical Parasitology (AIMP) malaria laboratory practice.

The final examination for the AIMP course consisted of 2 prompts, from which students selected one for data analysis. The first prompt involved training an AI model using malaria-infected and non-infected cell images from the United States National Institutes of Health malaria dataset (Supplement 2), as described in [4], followed by the preparation of a report evaluating the model’s performance. The second prompt focused on detecting helminth eggs using deep learning techniques [5]. I chose the malaria parasite detection task as my final exam topic. Teachable Machine, AI Studio, and vibe coding tools facilitated the development of the required AI model and proved effective in differentiating novel malaria-infected blood cell images that were not included in the initial training dataset. For example, the AI model generated in AI Studio classified a microscopic image of a *Plasmodium vivax*–infected sample with 95% confidence (Fig. 2). Although the examination required only the development of a relatively simple AI model, the exercise underscored the potential of such tools for future parasitology research requiring more refined differentiation and classification (Supplements 3, 4).

## Effectiveness of AI in learning

As someone with a bachelor’s degree in biology and a minor in AI from Case Western Reserve University, I was astonished by AI’s ability to generate functional code and practical content. I vividly recall spending a great deal of time and effort coding in undergraduate programming courses. With AI Studio vibe coding, programming no longer felt like a time-consuming burden; instead, AI could instantly generate code that met the criteria specified in the task bar. Beyond coding, AI also created content such as webpages, applications, and diagrams. For example, when instructions specifying user needs were provided, Antigravity (https://antigravity.google/) generated draft HTML pages that could be iteratively refined. By inputting National Center for Biotechnology Information (NCBI) accession numbers, the AI tool also generated phylogenetic tree diagrams of malaria-associated parasites based on published data. Antigravity was capable of iterative refinement, self-detection of coding errors, and program updates, which was particularly beneficial for users with little to no experience debugging code. Overall, AI tools such as Teachable Machine, AI Studio, and Antigravity lowered barriers to content development and demonstrated strong potential as valuable resources for future applications.

## Limitations of AI in the course curriculum

Despite its convenience, a curriculum focused heavily on AI implementation also had notable drawbacks. One major limitation stemmed from technical issues associated with AI tools. First, AI algorithms do not always generate accurate information; as the technology is still evolving, outputs may include false statements or irrelevant details, requiring expert validation and careful reevaluation. On several occasions, the AIMP professor paused student presentations to point out that certain AI-generated content presented in class was inaccurate or not applicable.

Technical limitations also negatively affected learning efficiency. Some AI tools failed to retain user prompts, necessitating repeated inputs and leading to frustration and lost time. Server congestion further restricted access, particularly when many students attempted to use the same platform simultaneously. As a result, considerable class time was occasionally devoted to troubleshooting rather than engaging with the course material. Performance disparities were also observed among AI platforms, with some tools outperforming others depending on the prompt and input format. In the 2023 AIMP course, students reported that generative AI produced different results when queries were entered in Korean versus English [6]. Given these limitations, AI should function as a supplementary learning aid rather than a primary source of information. Traditional resources, such as textbooks and peer-reviewed literature, remain essential for acquiring professional knowledge.

## Future of education implementing AI

I am confident that the AIMP course represents the beginning of a new educational era in which AI is used as a central tool to enhance understanding and retention of course material. Given the rapid pace of technological advancement, AI-implemented curricula are likely to become increasingly common in academic settings. I have little doubt that a variety of AI tools will remain an integral part of my medical education and will continue to influence my professional career beyond medical school.

## Supplementary materials

Supplement 1. The syllabus for the AIMP course at Hallym University provided to students before the start of class.

Supplement 2. The image dataset used for deep learning training on malaria parasite detection was selected from the dataset used by Reddy et al. [4].

Supplement 3. The author’s answer to the AIMP final exam prompt on malaria parasite detection.

Supplement 4. TensorFlow Lite model for deep learning training on malaria parasite detection.

## References

[1] Delello JA, Sung W, Mokhtari K, Hebert J, Bronson A, De Giuseppe T (2025). AI in the classroom: insights from educators on usage, challenges, and mental health. Educ Sci.

[2] Xu X, Chen Y, Miao J (2024). Opportunities, challenges, and future directions of large language models, including ChatGPT in medical education: a systematic scoping review. J Educ Eval Health Prof.

[3] Kang J, Ahn J (2025). Technologies, opportunities, challenges, and future directions for integrating generative artificial intelligence into medical education: a narrative review. Ewha Med J.

[4] Reddy CK, Anisha PR, Almushharaf A, Talla R, Baili J, Cho Y, Nam Y (2025). An optimized transfer learning approach integrating deep convolutional feature extractors for malaria parasite classification in erythrocyte microscopy. Front Med (Lausanne).

[5] Mirzaei O, Ilhan A, Guler E, Suer K, Sekeroglu B (2025). Comparative evaluation of deep learning models for diagnosis of helminth infections. J Pers Med.

[6] Lee H, Park S (2023). Information amount, accuracy, and relevance of generative artificial intelligence platforms’ answers regarding learning objectives of medical arthropodology evaluated in English and Korean queries in December 2023: a descriptive study. J Educ Eval Health Prof.

