# Supplementary material for: Implementation of artificial intelligence in the 2025 medical parasitology course at Hallym University
Source: J Educ Eval Health Prof. 2026 Feb 5;23:4. doi: 10.3352/jeehp.2026.23.4 (PMC12976625; doi:10.3352/jeehp.2026.23.4)
Supplement: Supplementary file 1 — Supplement 1. The syllabus for the AIMP course at Hallym University provided to students before the start of class. [file jeehp-23-04-suppl1.pdf]

2025학년도 의학과
제 3학년 (본1) 제 2학기

기생충학 수업계획서

조 번호:

계측렌즈 번호:

이름:

한림대학교 의과대학 기생충학교실

수 업 계 획 서

2025학년도 제 2학기

<table>
<tr><td>교과목명</td><td colspan="2">기생충학</td><td colspan="2">학점</td><td>인체와질병 II 에 포함</td></tr>
<tr><td rowspan="3">담당교수</td><td rowspan="2">성명</td><td rowspan="2">허 선</td><td colspan="2">연구실</td><td>의학관 3504</td></tr>
<tr><td colspan="2" rowspan="2">면담가능시간</td><td rowspan="2">화요일 오전 10-12시
그 외 수시로 가능</td></tr>
<tr><td rowspan="2">연락처</td><td rowspan="2">033-248-2652</td></tr>
<tr><td></td><td colspan="2">전자우편</td><td></td></tr>
<tr><td>강의목적</td><td colspan="5">수강자는 일차 진료에 필요한 기생충질환의 진단 및 치료에 대한 지식과 수기를 익히고 적용할 수 있어야 한다.</td></tr>
<tr><td>강의개요</td><td colspan="5">우리나라의 기생충질환에 대한 생물학, 역학, 진단, 치료 및 예방법에 대한 기본지식을 이해할 수 있도록 설명한다.</td></tr>
<tr><td>강의방법</td><td colspan="5">강의, 토의, 질의응답, 학생 발표, 실습, 생성형 AI, deep learning 등 다양한 방법 동원</td></tr>
<tr><td>교 재</td><td colspan="5">임상기생충학(서울대학교출판문화원)</td></tr>
<tr><td>참고도서</td><td colspan="5"></td></tr>
<tr><td rowspan="5">평가방법
(100%)</td><td>Computer-Based Testing</td><td>30 %</td><td rowspan="5">※ 출석미달 기준 등</td><td colspan="2" rowspan="5">전체 시간의 90% 이상 출석하지 못할 경우 미달로 성적이 나가지 않음.
흡연자는 수업참여 불가 및 시험 참여 불가
별도 성적이 나가지 않고 Pass/Fail 로 평가함</td></tr>
<tr><td>개인준비도측정</td><td>10 %</td></tr>
<tr><td>TBL/PBL 동료 평가</td><td>20 %</td></tr>
<tr><td>출 석</td><td>10 %</td></tr>
<tr><td>실습시험
(학명시험 포함)</td><td>30 %</td></tr>
</table>

※ 총 수업시간: 강의 14시간, 실습 28시간, 시험 3 시간 계 51 시간

월	일	교시	구분	강의내용 (실습내용)	교실명	교수명
10	27	1,2	강의	기생충과 기생생활, 장내 선충증	기생충학교실	허선<br>이윤환
		6,7<br>8,9	실습	실습, 충란 계측법 및 장내 선충증	〃	〃
11	3	3,4	강의	조직내 선충증 (온라인)	〃	〃
		6,7<br>8,9	실습	실습, 조직내 선충증	〃	〃
11	10	3,4	강의	흡충증	〃	〃
		6,7<br>8,9	실습	실습, 흡충증	〃	〃
11	17	1,2	강의	조충증	〃	〃
		3,4	강의	말라리아	강원대	한은택
		6,7<br>8,9	실습	실습, 말라리아	〃	한은택<br>이윤환
11	19	6,7<br>8,9	실습	실습, 조충증	기생충학교실	허선<br>이윤환
12	1	1,2	강의	원충증	기생충학교실	허선<br>이윤환
		6,7<br>8,9	실습	실습, 원충증	〃	〃
12	5	3,4	강의	절지동물감염증<br>Chemotherapy of parasitic diseases	〃	〃
		6,7<br>8,9	실습	실습, 절지동물감염증	〃	〃
12	15	2,3<br>4	시험	CBT, 실습시험	〃	〃

대안렌즈 눈금	대물렌즈			
	× 4	× 10	× 40	× 100
1				
2				
3				
4				
5				
6				
7				
8				
9				
10				
11				
12				
13				
14				
15				
16				
17				
18				
19				
20				

학생실습용 슬라이드 2025학년도 제 2학기 한림의대 제 1학년

1. *Ascaris lumbricoides* female, esophageal level, H&E
2. *Ibid*, female, midgut level, H&E
3. *Ibid*, male, hindgut level, H&E
4. *Trichuris trichiura*, male
5. *Ibid*, female
6. *E. vermicularis* female
7. *Strongyloides stercoralis*
8. Anisakis type Ⅰ
9. Stomach anisakiasis from human, H&E
10. Anisakis type Ⅰ in rat stomach, H&E
11. *Trichinella spiralis* larva in mouse muscle
12. *Clonorchis sinensis* from experimental rabbit, AC
13. *Paragonimus westermani*
14. *Schistosoma japonicum* adult from exp. mice
15. *Metagonimus takahashii* from man, AC
16. *Echinostoma hortense* from experimental rat, AC
17. *Diphyllobothrium latum* from man, gravid proglottid, AC
18. *Taenia saginata*, gravid proglottid, AC
19. *T. solium*, gravid proglottid, AC
20. *Spirometra erinacei*, mature proglottid, AC
21. Cysticercus cellulosae from pork whole mount
22. Cysticercus cellulosae in pork
23. *Echinococcus granulosus*
24. *Plasmodium falciparum*
25. *Plasmodium falciparum* from culture, Giemsa
26. *P. vivax* from Singapore, Giemsa
27. *Eimeria stiedae* in rabbit bile duct, H&E
28. *Trichomonas vaginalis* from culture
29. *Toxoplasma gondii* trophozoite from mouse peritoneum, Giemsa
30. *Entamoeba histolytica* in caecum, H&E
31. *Leishmania major* from culture, H&E
32. *Cryptosporidium* from experimental mouse, Giemsa
33. *Pediculus humanus* var. capitis
34. *Phthirus pubis*
35. *Leptotrombidium pallidum* from a field mouse, caught in Pachu
36. *Giardia lamblia* Trophozoite
37. *Schistosoma mansoni* in mouse intestine
38. *Capillaria hepatica* in mouse liver

# - 차례 -

수업계획서	2
학생실습용 슬라이드	5
1. 교과목이름	7
2. 학점	7
3. 담당교원	7
4. 대상학년 학기 및 수강생 수	7
5. 학습목표	7
6. 수업진행 방법	7
7. 학습평가 방법	8
8. 주요교재 및 참고교재	8
9. 각 단위별 교과 진도 계획 및 내용	8
10. 수업 종강시의 수업에 대한 수강자의 반응 및 평가 요령	16
11. 기타 수업의 효율성 제고를 위한 항목	16
실습의 학습 목표와 구체 내용	17
생성형 인공지능 플랫폼과 deep learning 활용한 자율학습	28
팀바탕학습(Team-based learning)	29
문제바탕학습(Problem-based learning)	34

1. 교과목이름: 기생충학(전공 필수)

2. 학점: 인체와 질병 II 에 포함(강의 14시간, 실습 28시간, 시험 3시간, 총 45시간)

3. 담당교수: 허 선(기생충학교실)

외래교수: 한은택(강원의대), 이윤환(한림대학교 시간 강사)

4. 대상학년 학기 및 수강생 수: 의학과 제 1학년 제 2학기, 76 명

5. 학습목표: 일차 진료에 필요한 기생충질환의 진단 및 치료에 대한 지식과 수기를 익히고 적용할 수 있어야 한다.

6. 수업진행 방법

강의 또는 학생 스스로 생성형 AI platform, deep leariningà을 활용한 자기 주도 학습을 통하여 기생충질환의 학문성과를 이해하고, 그 중에서도 우리나라 또는 온누리에서 중요한 질환에 대한 접근을 실습을 통해 직접 시도한다.

1) 강의: Team-based learning, problem-based learning, 교탁 강의

2) 실습: 실습은 실습 시작하자마자 내용에 해당하는 비디오 프로그램을 약 15-20분간 상영하여, 실습 전 실험실에서 준비 과정과 실습 과정을 보여 준다. 즉 모의 과정(simulation)을 미리 익히게 한다. 그리고 그날의 유리 슬라이드 표본과 맨눈 표본을 보도록 하고, 동물 실험 또는 검사도 병행하여, 각 조별로 하도록 한다. 실습은 한 조를 10~11명으로 구성하여 8조로 나누어 나름대로 진행하도록 한다. 실습 도중의 질문은 전임교원이 실습실에서 답해 주고, 학생들이 관찰한 것이 정확한 것인지 확인한다. 실습 진행 4시간 동안 자유롭게 시간 나는 대로 휴식을 취하면서 진행하도록 한다. 가능한 한 실습 과정을 학생들이 직접 하도록 한다. 실습용 슬라이드 표본은 한 조에 하나씩 학기 시작하면서 박스에 담아 나누어준다. 맨눈 표본은 매시간 전시한다. 슬라이드 수가 각 조별로 나누어주기 부족한 것은 전시 슬라이드로 보게 하고, 그마저 없는 표본은 프로젝트용 슬라이드로 보여 준다. 동물실험 도구로 조직감자(tissue forceps), 가위는 각 두개씩을 한 조에 학기 초에 나누어준다. 나누어준 표본과 기구는 시험 치른 뒤 반환하도록 한다. 실습 매시간 관찰한 내용은 실습책에 그려 충체와 구조의 이름을 쓰고 크기를 적도록 한다. 실습책은 매주 회수하여 잘못 표기한 것을 지적하여 돌려준다.

* 주의: [실습 슬라이드 및 가위, 포셉의 분배 및 회수] 학기 시작할 때, 슬라이드 상자 위에 조원의 이름을 다 쓰고, 마지막 실습 시간에 모두 회수한다.

7. 학습평가 방법

1) 배점: CBT 30 %, 학명시험을 포함한 실습시험이 30 %, 개인 준비 측정 10%, TBL/PBL 동료 평가 20%, 출석 10 % 로 배당한다.

2) CBT 문항의 유형: 선다형 문제(multiple choice)로 하고, 누리그물 이용하여(internet-based testing) 셈틀기반검사(computer-based testing)를 치른다.

3) 성적 산정: 인체와 질병 II에 포함하나 Pass or Fail 로 별도 성적을 제출하지 않는다.

4) 결석에 대한 점수 제함: 특별한 사유 없는 결석은 강의나 실습 한 시간당 총 100점 만점에서 1 점을 제한다. 출석 점수 제하는 것이 10 점을 초과할 때는 시험 응시 자격을 주지 않는다. 결석이나 지각할 때는 바로 집에 알려서 문제가 있는지 확인한다.

5) 재시: CBT 재시는 시험성적이 일정 수준 이하인 경우 시행할 수 있다. 일정기준 도달할 때까지 반복

6) 성적에 이의 신청: 시험지는 공개하지 않으므로 성적 이의 신청자는 개별 방문하여야 한다.

7) 성적공개: 시험을 치른 후 곧 성적을 공개하고, 재시 후의 성적도 재시 치른 뒤 곧 공개한다.

8) CBT, 실습 시험 날짜 지정: 개인준비 측정 시험을 치르고, 기말시험을 치른다.

9) 실습시험: 사전에 실습 시험 문항을 제공하므로 해당하는 기생충란, 충체 현미경 소견과 맨눈 소견을 익혀야 한다. 30초마다 자리 이동하는 땡시험으로 치룬다. 문항수는 20개이다. 각자 10분이면 땡시험을 마친다.

8. 주요교재 및 참고교재

주요교재 - 임상기생충학. 서울대학교출판문화원

참고교재 - PubMed and KoreaMed, PubMed Central, Mediscape, Gitbub

9. 각 단위 별 교과 진도 계획 및 내용

수업의 구체 내용(학습목표)은 아래와 같다. 강의의 구체 학습 목표는 의과대학학장협의회 학습목표(2007)에서 학교 실정에 맞게 수정하여 만들었다.

제 1 주 기생충과 기생생활, 장내 선충증

1) 기생충학을 정의할 수 있어야 한다.
2) 숙주와 기생충 상호관계의 특이성을 설명할 수 있어야 한다.
3) 이명법으로 학명을 기술할 수 있어야 한다.
4) 인체 기생충을 전파양식에 따라 분류하고 예를 들 수 있어야 한다.
5) 우리나라에서 발견된 인체 기생충을 원충, 선충, 흡충, 조충, 절지동물별로 나열할 수 있어야 한다.
6) 기생충의 일반 진단과 치료, 예방법을 설명할 수 있어야 한다.
7) 윤충을 세 가지 강(선충, 흡충, 조충)으로 나눌 수 있어야 한다.
8) 윤충의 일반 특징을 설명할 수 있어야 한다.
9) 선충의 일반 특징을 설명할 수 있어야 한다.
10) 장내 선충증의 역사, 형태, 생활사, 병리 및 증상, 진단, 치료, 역학, 예방 및 관리 등을 기술할 수 있어야 한다.
11) 장내 선충의 국내 감염 예를 2 가지 이상 나열할 수 있어야 한다.

제 2주 조직내 선충증

1) 조직내 선충증의 역사, 형태, 생활사, 병리 및 증상, 진단, 치료, 역학, 예방 및 관리 등을 설명할 수 있어야 한다.
2) 조직 내 선충의 국내 감염 예를 두 가지 이상 나열할 수 있어야 한다.

제 3주 흡충증

1) 흡충의 일반 특징을 설명할 수 있어야 한다.
2) 우리나라에 토착 인체 기생 흡충증을 나열할 수 있어야 한다.
3) 혈관에 기생하는 주혈흡충을 기생부위에 따라 3가지로 나누어 열거할 수 있어야 한다.
4) 우리나라에 주혈흡충증 수입례 보고된 증례의 감염 장소를 열거할 수 있어야 한다.
5) 간흡충 감염 시, 증상, 병변, 합병증을 설명할 수 있어야 한다.
6) 간흡충 치료제를 들고, 그 치료제의 보급에도 불구하고 간흡충증의 유병률이 급속히 감소하지 않고 있는 현상을 주민 행태 측면에서 설명할 수 있어야 한다.
7) 간흡충증과 담도상피암과의 상관관계를 설명할 수 있어야 한다.
8) 우리나라 폐흡충증의 국소 유행지를 나열할 수 있어야 한다.
9) 폐흡충증의 면역 진단 방법을 설명할 수 있어야 한다.
10) 폐흡충증 이소기생 부위 및 증상을 설명할 수 있어야 한다.
11) 우리나라에 토착감염으로 보고된 장흡충을 10개 이상 나열할 수 있어야 한다.
12) 장흡충증의 진단, 치료법을 설명할 수 있어야 한다.

제 4주 조충증

1) 조충의 일반 특징을 설명할 수 있어야 한다.
2) 조충의 발육단계, 형태 특징을 설명할 수 있어야 한다.
3) 광절열두조충, 만손열두조충의 형태 감별, 생활사, 감염 시 증상을 설명할 수 있어야 한다.
4) 고충증의 진단법을 3가지 이상 나열할 수 있어야 한다.
5) 고충의 인체감염 시 면역회피 기전을 설명할 수 있어야 한다.
6) 유구조충, 무구조충, 아시아조충의 형태 감별, 생활사, 감염 시 증상을 설명할 수 있어야 한다.
7) 조충의 조충애벌레 감염증(유구낭미충증, 고충증, 포충증)이 성충 감염 때보다 심한 병변을 인체에서 일으키는 이유를 설명할 수 있어야 한다.
8) 조충애벌레 감염증의 진단, 치료법을 설명할 수 있어야 한다.
9) 조충 성충의 인체 감염 때 치료제를 한 가지 들 수 있어야 한다.

제 5주 말라리아

1) 인체 기생 학질 원충 4가지를 나열할 수 있어야 한다.
2) 인체 기생 학질 원충 4가지의 현미경 감별 점을 설명할 수 있어야 한다.
3) 우리나라에 1960년대 말까지 유행한 삼일열원충증이 소멸한 이유와 1990년대 다시 재발한 이유를 설명할 수 있어야 한다.
4) 새로운 학질 약제 개발의 필요성을 설명할 수 있어야 한다.
5) 학질 유행지 여행할 때, 예방법을 설명할 수 있어야 한다.

제 6주 원충증

1) 원충이 포낭을 형성하는 합목적성을 설명할 수 있어야 한다.
2) 람블편모충이 지방변을 일으키는 기전을 설명할 수 있어야 한다.
3) 이질아메바의 병변과 이소기생 부위를 나열할 수 있어야 한다.
4) 장내 원충증의 치료법을 설명할 수 있어야 한다.
5) 질편모충의 진단 및 치료법을 설명할 수 있어야 한다.
6) 병원성 자유생활아메바의 인체 감염 경로와 감염 때 증상을 설명할 수 있어야 한다.
7) 리슈만편모충과 파동편모충 감염증 가운데 우리나라에 수입례가 보고된 것에 어떤 것이 있는지 나열할 수 있어야 한다.
8) 두 가지 아프리카 수면병의 임상상을 구분하여 설명할 수 있어야 한다.
9) 작은와포자충, 사람등포자충, 원포자충속의 역학, 진단 및 치료법을 설명할 수 있어야 한다.
10) 톡소포자충증의 면역타협환자에서 임상상을 설명할 수 있어야 한다.

제 7주 의용절지동물학

1) 의용절지동물학을 정의 내릴 수 있어야 한다.
2) 의용절지동물의 임상 중요성을 매개체로서와 직접병변을 일으키는 기생체로서 설명할 수 있어야 한다.
3) 사람에 기생하는 이의 종류를 나열할 수 있어야 한다.
4) 이의 구제법을 설명할 수 있어야 한다.
5) 옴의 증상, 진단, 치료법을 설명할 수 있어야 한다.
6) 쯔쯔가무시병 매개 진드기의 학명을 댈 수 있어야 한다.
7) 먼지진드기와 알레르기와의 관계를 설명할 수 있어야 한다.

10. 수업 종강시의 수업에 대한 수강자의 반응 및 평가 요령

2002년부터는 학교 누리그물 설문을 사용한다.

11. 기타 수업의 효율성 제고를 위한 항목

의과대학의 수업은 대부분 교실 단위 수업이다. 즉, 한사람의 교원이 한 학기에 한 강좌를 개설하여 이끄는 것과 다르게 한 과목을 한 교실에서 맡아 수업을 한다. 그러므로 여러 교원이 참여하고, 실습도 여러 전임교원과 조교가 참여한다. 그러나 우리 학교 기생충학교실에는 아직 전임교원이 한 명, 조교가 한 명이므로 책임 소재가 매우 분명하다. 그런 반면, 적은 인원의 한계를 극복하기 위하여 우리나라 기생충학 분야의 우수한 다른 대학 교원을 초빙하여 그 분야에 대하여 더 깊이 있는 강의를 학생들이 듣도록 배려하였다. 그러한 외래교수의 초빙은 앞으로 졸업생들이 실제 임상에 나가, 전국 어디에 근무하더라도 접할 수 있는 전문가이기에 학부 때에 얼굴을 익히는 것도 평생 자문을 얻어야 한다는 점에서 매우 중요하다. 학습 목표 달성을 위하여 어떻게 접근하여야 하는 데에서, 교실원과 학생들 사이의 관계 형성을 근본으로 하여 수업계획을 작성하였다. 그러기 위해서는 기생충학이 재미있고, 기생충학자는 접근하기 쉽다는 인상을 심어 주어야 한다. 가능하다면 최소한의 지식과 수기를 익히고, 그 이상의 것은 언제든지 전문가의 자문을 얻는 자세, 책을 찾고, 잡지를 찾아보는 자세를 갖도록 준비하였다. 9편의 비디오를 자체 제작한 것도 가능하다면 조금 더 흥미를 끌 수 있도록 하기 위하여 준비하였다.

= 실습의 학습 목표와 구체 내용 =

강의 수업과 연관되게 계획하였으나 추구 관찰이 필요한 동물실험은 일부 수업보다 미리 또는 나중에도 실시하도록 조정하였다.

실습의 일반 목표: 강의에서 들은 내용을 실제 표본으로 관찰하고, 직접 동물실험을 통해, 주요 토착 기생충질환의 생활사, 진단, 치료법을 직접 실습을 통해 설명할 수 있어야 하고, 또한 우리나라에는 수입병으로 보고되거나 보고 안 된 기생충병의 원인 기생충도 체내, 체외 배양을 통하여 실습에서 다루어 세계의 질병을 이해할 수 있어야 한다.

실습할 때 학생의 준비

1. 실험복을 반드시 입어야 한다. 자신을 보호하고 옷에 오염물질이 묻는 것을 막기 위하여서이다.
2. 실습 시간에 교과서를 항상 준비하여야 한다.
3. 매 시간에 눈금렌즈(ocular micrometer)를 나누어주므로 자신의 번호의 렌즈를 가져갔다가 실습 끝날 때 제자리에 꽂아 놓아야 한다.
4. 매주 돌아가면서 한 조씩 실험 준비물 운반과 뒷정리를 하여야 한다. 실습 시간 전후로 조교가 일러주는 조가 담당 조이다. 특별한 예외가 없는 한 조별로 차례로 진행된다. 실습 10분 전에 교실(3504호)로 와서 실습 준비물을 날라야 한다.

찰흙을 이용한 기생충 만들기

기생충의 구조를 이해하고 각 조마다 천연색 찰흙을 이용하여 기생충 모형을 제작하여 형태를 익힌다. 준비물은 천연색 찰흙이며 각 조마다 실습 시작할 때 제공한다.

제 1주

1-1. 현미경 계측

가. 학습목표: 현미경 시야에서 표본의 길이를 측정하는 법을 알아야 한다.
나. 준비물: 현미경, 계측 접안렌즈(100구간의 눈금이 새겨져 있다), 계측용 슬라이드(중앙에 1 ㎜를 100 등분하여 한 눈금이 정확히 0.01 ㎜인 자이다), 계산기
다. 사용 방법: 눈금 달린 대안렌즈를 사용할 때는 왼쪽의 대안렌즈를 돌려서 빼고 눈금 달린 대안렌즈를 넣어야 한다.

현미경 계측법

1) 왼쪽 대안렌즈에 계측 렌즈를 장치한다.
2) 현미경의 재물대에 계측용 슬라이드를 끼워 넣는다. 이때 앞뒤를 잘 구분하여 넣는다.
3) 계측(대물)렌즈를 저배율로 고정시킨 뒤, 초점을 맞추고 움직여서 시야의 계측용 슬라이드의 눈금과 일치하도록 조절한다.
4) 두 눈금이 정확히 일치하는 곳의 계측렌즈의 눈금 수와 계측용 슬라이드의 길이를 읽은 후, 비례식으로 계측 렌즈의 한 눈금의 길이를 계산한다.
5) 같은 작업을 배율을 바꾸어 가면서 시도한다.

예를 들어 대안렌즈 배율이 4배일 때, 계측렌즈의 눈금 22개가 계측용 슬라이드 눈금 50개와 일치한다고 치자.

그럴 경우 계측 렌즈 한 눈금에 해당하는 길이는
50 × 0.01 ㎜ / 22 = 0.0227 ㎜ = 22.7 ㎛

즉, 다음부터 대안렌즈 4배(현미경 전체에서는 40배)의 배율에서 대안렌즈 한 눈금에 해당하는 표본의 길이는 언제나 22.7 ㎛ 이다. 이 계측 렌즈는 남의 것과 바꾸어 쓰지 말아야 한다. 대개 현미경의 렌즈는 성능이 비슷하나 조금씩 다를 수 있기 때문이다.

○ 윤충란의 크기 및 특징

	크기(micrometer)	특 징
1) *A. lumbricoides* (F)	50-75 × 35-50	둥근 타원꼴, 갈색, 3층의 난각 (돌기모양의 단백질피막, 키틴피막, 유지방체층)
(U)	80-90 × 45	길쭉한 타원꼴, 갈색, 얇은 난각, 불규칙한 단백질피막
2) *T. trichiura*	50-54 × 20-23	술통꼴, 갈색, 양극의 점막전
3) *C. sinensis*	27-35 × 12-20	길쭉한 달걀꼴, 밝은 황갈색, 뚜렷한 shouldering, muskmelon 꼴 난각, 미라시디움 포함
4) *M. yokogawai*	28-30 × 16-17	달걀꼴, 황갈색, 미끈한 난각
5) *D. latum*	66-75 × 45-53	넓은 달걀꼴, 갈색, 매끈하고 얇은 난각

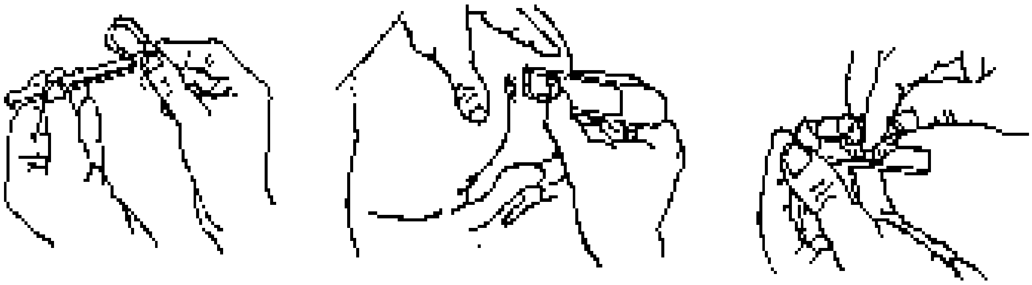

그림 1. 셀로테이프 검사법. 그림은 Brown & Neva, Basic Clinical Parasitology 5th ed. p.131

1-2 윤충란 동정, 장내 선충증

가. 학습 목표

① 현미경으로 충란을 관찰하는 법을 알아야 한다.

② 각 충란의 특징을 기술할 수 있어야 한다.

③ 충란과 이물질을 구별할 수 있어야 한다.

④ 회충, 편충, 요충의 맨눈 표본과 조직 표본을 감별할 수 있어야 한다.

⑤ 요충 검사법으로 셀로테이프법을 시행할 수 있어야 한다.

나. 표본

* 충란현탁액병(회충, 편충, 간흡충, 요코가와흡충, 광절열두조충, 만손열두조충)

: 대변을 포르말린에테르 집란법으로 처리하여 충란을 모아 10 % 포르말린에 저장한 것임.

* 슬라이드 표본 번호: 1-7 (번호는 5쪽에 내용이 수록)

다. 맨눈 표본: *Ascaris lumbricoides*, M & F

Trichuris trichiura, M & F

Enterobius vermicularis, F

라. 관찰방법

① 현탁액병을 흔든 후, 막대를 두 개 넣어 현탁액 한 방울을 슬라이드글라스에 떨어뜨린다.

② Lugol's iodine 한 방울을 그 위에 떨어뜨린 뒤, 막대로 젓는다.

③ 커버슬립을 덮은 뒤, 현탁액이 흘러넘치면 여과지로 흡수시킨다.

④ 100배 시야에서 충란을 찾고, 400배 시야에서 충란의 자세한 형태를 관찰한다.

⑤ 각 충란의 형태를 그리고, 길이와 폭을 표시하고, 각각의 특징 있는 구조물을 써넣는다.

마. 비디오: 선모충

내용은 선모충을 실험 감염된 마우스 근육에서 회수하여, 다시 새 마우스에 감염시킨 뒤 한 달 지나 근육에서 회수하는 것으로 감염된 동물근육의 생식이 감염경로 임을 보여 준다.

바. 준비물

현미경, ocular micrometer, stage micrometer, 슬라이드글라스, 커버슬립(22㎜×22㎜), 막대, 여과지, 렌즈페이퍼, 포셉, Lugol's iodine, 비디오테이프

제 2주 조직내 선충

가. 학습 목표:

① 고래회충 유충과 선모충유충의 맨눈 소견과 조직 소견을 다른 선충의 것과 감별할 수 있어야 한다.

② 고래회충 유충의 붕장어에서 기생부위를 확인하고 골라낼 수 있어야 한다.

나. 슬라이드 표본 번호: 8-11, 38

다. 맨눈 표본: *Anisakis simplex* type I larva

라. 전시 슬라이드

Thelazia callipaeda

Microfilaria of *Brugia malayi*

마. 비디오

고래회충 유충 - 내용은 실습에서 직접 할 내용을 미리 보여주는 것이다.

간흡충 - 내용은 간흡충피낭유충에 감염된 참붕어를 소화시켜 흰쥐에 감염시킨 뒤 4주 후 회수하는 전 과정을 보여 준다.

바. 준비물: 유리슬라이드, ocular meter, 생리식염수, 붕장어, 붕장어 내장, 비디오테이프

사. 검사: 고래회충 유충

바다생선 중 꾀붕장어 (*Anago anago*)장간막에서 고래회충 유충을 골라서 관찰한다.

제 3주 흡충증

가. 학습목표

① 간흡충과 폐흡충의 맨눈표본, 조직 표본을 동정할 수 있어야 한다.

② 요코가와흡충, 호르텐스극구흡충, 서울주걱흡충의 형태를 동정할 수 있어야 한다.

③ 장흡충 감염 때, 장병변을 설명할 수 있어야 한다.

나. 슬라이드표본: 12-16, 37, 서울주걱흡충 adult, AC

다. 맨눈표본: 간흡충, 서울주걱흡충

라. 전시슬라이드:

1) Human liver infected with *Clonorchis sinensis,* H & E

2) *Paragonimus westermani,* adult worm, Acetocarmine

3) Intestinal metagonimiasis in rat

마. 검사 및 동물 실험

바. 비디오: 서울주걱흡충, 고충, 간흡충

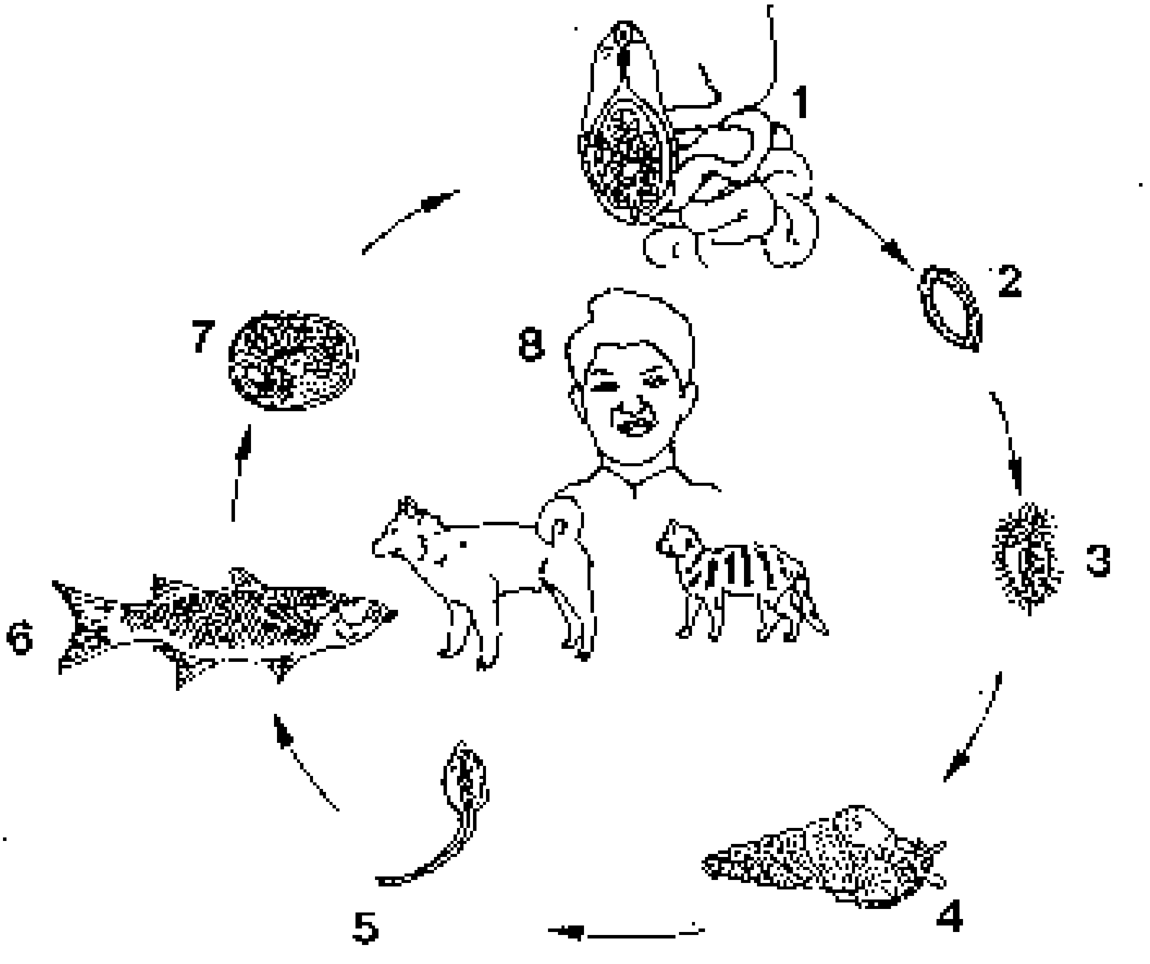

이형이형흡충의 생활사 설명

1. 숙주의 소장에 기생하는 성충이 충란을 배출한다.
2. 몸 밖으로 배출된 충란은 강물로 들어간다.
3. 물속에서 충란은 미라시디움으로 성장한다.
4. 이 미라시디움이 제 1 중간숙주인 담수산 패류에게 먹힌다.
5. 담수산 패류에서 스포로시스트, 레디아, 유미유충으로 성장하여 패류에서 다시 물속으로 나온다.
6. 유미유충은 제 2 중간숙주인 숭어와 같은 민물고기나 반염수어의 비늘에 붙은 다음 꼬리는 떨어지고 몸체만 근육 속으로 들어간다.
7. 제 2 중간숙주 체내에서 피포되어 피낭유충이 된다.
8. 이 피낭유충에 감염된 제 2 중간숙주를 날로 먹거나 충분히 익혀 먹지 않을 때, 사람, 개, 고양이 등의 숙주의 소장에 들어와 장점막에 기생한다. 숙주 소장의 장점막에서 다시 충란을 배출하여 생활사를 이어간다(저자 그림).

제 4주 조충증

가. 학습 목표

① 의엽조충을 맨눈으로 감별할 수 있어야 한다.

② 광절열두조충, 만손열두조충의 편절을 동정하고 감별할 수 있어야 한다.

③ 고충을 맨눈과 조직소견에서 동정할 수 있어야 한다.

④ 유구조충 편절을 동정하고, 감별할 수 있어야 한다.

⑤ 유구낭미충을 맨눈과 조직소견에서 동정할 수 있어야 한다.

⑥ 원엽조충과 의엽조충을 맨눈으로 감별할 수 있어야 한다.

나. 슬라이드 표본: 17-23

다. 맨눈 표본

Spirometra erinacei from cat

Sparganum (metacestode of *S. erinacei*) from snake

Taenia saginata

Metacestode of *T. solium* infection from pig

라. 전시슬라이드

Scolex of *S. erinacei*

제 5주 말라리아

가. 학습 목표

① 인체 감염 학질원충 중 열대열, 삼일열원충을 혈액 도말 표본에서 동정할 수 있어야 한다.

② 학질 원충의 발육단계를 구별할 수 있어야 한다.

나. 슬라이드 관찰: 24-26

다. 맨눈 표본: 없음

라. 전시 슬라이드: 없음

마. 검사 및 동물 실험

바. 비디오

사. 준비물

제 6주 원충증

가. 학습목표

① 원충포낭을 표본에서 동정할 수 있어야 한다.

② 질편모충의 운동을 관찰할 수 있어야 한다.

③ 리슈만편모충의 구조를 확인할 수 있어야 한다.

④ 톡소포자충의 tachyzoite를 동정할 수 있어야 한다.

⑤ 작은와포자충의 구조를 확인할 수 있어야 한다.

나. 슬라이드 표본: 27-32, 36

다. 원충 포낭 부유액 관찰: 병에 들은 원충 포낭을 면봉으로 떠서 슬라이드 위에 한 방울 얹고, 커버슬립으로 덮은 뒤, 현미경 400배 시야로 관찰한다. 루골 요오드로 염색하여 관찰한다.

* Iodide wet smear

준비물: 루골 요오드(iodine 1 g, KI 2 g, D/W 100 cc), 포르말린 에테르법의 준비물

방법: 포르말린-에틸아세테이트법으로 구한 표본을 슬라이드 위에 떨어뜨린 뒤,
루골 요오드를 한 방울 떨어뜨려 커버슬립을 덮고 관찰한다.

* 결과: oocyst: 색 없음, yeast: 갈색, 원충포낭: 갈색으로 염색되어 핵, 글리코겐 뚜렷이 볼 수 있다.

라. 배양된 질편모충 관찰: 시험관에 배양된 질편모충을 슬라이드 위에 얹고 커버슬립을 씌워 현미경 450 배, 1,000 배 시야에서 운동을 관찰한다. 편모의 운동을 관찰한다.

마. 비디오: 이질아메바, 리슈만편모충, 톡소포자충

이질아메바의 영향형의 운동을 보여 준다. *Leishmania major*의 운동 모습을 보여 준다. 톡소포자충 tachyzoite를 보여 준다.

바. 준비물: 원충포낭 부유병, 면봉, 배양된 이질아메바가 들은 시험관(연대), 질편모충이 들은 시험관(한양대), 수조(37℃), 루골 요오드액, 유리슬라이드, 커버슬립, 온도계, 받드, 생리식염수, 페트리디쉬, 스포이드

원충포낭의 감별

이질아메바: 12-20 ㎛, 달걀꼴, central karyosome, peripheral chromatin, 4 nuclei

대장아메바: 10-33 ㎛, 원형, eccentric karyosome, 1-8 nuclei

소형아메바: 8-10 × 4-5 ㎛, 1-4 nuclei

람블편모충: 4-21 × 5-15 ㎛, 달걀꼴, 2-4 nuclei, fibril, axoneme, parabasal body

제 7주 의용절지동물

가. 학습목표

① 머릿니, 사면발이를 동정할 수 있어야 한다.

② *Leptotrombidium pallidum*의 구조 특징을 관찰할 수 있어야 한다.

③ *Demodex folliculorum*을 안면에서 관찰하는 방법을 시행할 수 있어야 한다.

④ 집먼지진드기를 추출하여 관찰할 수 있어야 한다.

나. 슬라이드 표본: 33-35

다. 검사 및 동물 실험: *Demodex folliculorum* 관찰

① 실습 시간 시작할 때, 셀로테이프를 턱, 뺨, 콧등에 붙인다.

② 2 시간 후 테이프를 슬라이드글라스 위에 붙여 100배로 관찰한다.

③ 준비물: 슬라이드글라스, 셀로테이프

라. 집먼지진드기 관찰

① 수집한 집먼지진드기를 슬라이드 위에 놓고 관찰할 수 있어야 한다.

생성형 인공지능 플랫폼과 deep learning 활용한 자율학습

1. 모든 학습 목표는 Open AI 사 GPT 최신 버전으로 문의하여 자세한 답을 듣는다.
2. GPT 최신 버전의 답이 정확한지 문헌을 찾아서 확인하여, 관련성, 정확도를 정리한다.
3. Deep learning을 통하여 기생충관련 자료를 Google colab 통하여 훈련시킨다. 코드와 자료는 Gitbhub 에 공개된 것을 사용하고 학습자가 직접 촬영한 자료를 가지고 타당성을 판단한다.
4. 구체적인 방법은 강사 지도와 GPT를 활용하여 익힌다.

팀바탕학습 (Team-Based Learning)

**대규모 학생을 대상으로 하는 전통적인 수업 상황에서 소규모 활동의 효과성을 살릴 수 있는 방법**
수업 전 학생들로 하여금 자신이 학습해야 할 목표를 명확히 제시하고, 자신의 학습과정은 자기가 형성해 간다는 책임감을 부여하며, 그룹 토론에 참여할 준비를 확실히 할 수 있게 하며, 학생들로 하여금 참여를 유도할 수 있는 지침을 제공해 준다.

⇩

TBL의 효과적이고 능동적인 학습을 위한 3가지 중요한 요소

학습에 대한 개인과 소집단의 책임감	그룹 간의 상호작용의 필요성과 기회 제공	주고받기 식의 토론의 참여나 동기부여

≜ 진행 방식

7~8명 정도의 작은 그룹으로 나누어 진행하는 소그룹 활동

\+

전체 학생이 동시에 참여하는 활동

≜ 수업 진행 단계

**1단계**	**수업 시작 전, 설정된 학습목표에 따라 사전과제를 미리 부여하고 공부해 오도록 하는 과정**

⇩

**2단계**	**사전과제를 잘 수행하였는지 학습한 내용을 확인하는 과정**<br>① 다지선다형(객관식) 개인시험(IRAT; individual assurance readiness test) 실시<br>② 소집단 편성 - 개인시험과 동일한 문제를 소집단 별로 토론하여 공동의 의견 도출<br>③ 공동의 의견에 입각하여 다시 시험문제(GRAT; group assurance readiness test)를 보고 소집단 간 토론 실시<br>④ 소집단 활동에 의한 시험 성적에 의하여 소집단 별로 점수 산정

⇩

**3단계**	**1~2단계를 통하여 습득한 지식을 바탕으로 학습한 것들을 적용하는 단계**<br>☑ 경우에 따라서 연속적으로 몇 번에 걸쳐 진행 가능<br>☑ 전체 학생을 대상으로 하는 강의가 포함될 수 있고, 모든 그룹이 동시에 자신들의 해결방안을 비교하고 토론하는 장을 마련할 수도 있음<br>☑ 이때 교수는 그룹 간에 토론의 조정자 역할을 하며 학습이 일어날 수 있도록 역할을 해야 함

오클라호마대학의 미켈슨 교수의 효과적인 그룹운영을 위한 세 가지 요소

소그룹 수업방법은 여러 가지 장점을 갖고 있다. 그러나 이것은 소규모 그룹의 역동성과 학생들이 수업 전 충분히 학습을 위한 준비를 한다는 것을 전제로 하고 있다. 다행스럽게도 앞에서 열거한 세 가지 요소를 적용함으로써 대부분이 학습자 그룹에서 이러한 조건을 충족시킬 수 있다.
부연 설명을 하자면,

(1) 개인 그리고 집단으로서의 책임감을 고양시킨다.
(2) 수업 전 과제물을 통하여 개인, 그룹, 전체 그룹 상호 간의 토론을 유도 할 수 있다.
(3) 소규모 그룹 내 그룹 간의 주고받는 상호 작용의 방법을 배우게 된다.

특히 소그룹 수업에 있어서 최고의 결과를 얻기 위해서는 반드시 이 세 가지 요소를 전제해야 한다.

책임감의 고양

만일 학생들 모두가 소규모 수업을 위한 준비를 하지 못했을 때에는 소규모 그룹으로 하는 수업자체가 수업 준비를 잘 해온 준비된 학생들이 준비를 하지 않은 학생들을 이끌어 갈 수 있도록 해야 한다. 그러나 제대로 준비되지 못한 소규모의 수업은 결국 배움의 발생보다는 일종의 사교 모임으로 되고 말 것이다. 이를 피하기 위해서는 사전에 과제물을 내주고 각 개인이 자기 학습을 통해서 소규모 그룹에 대한 책임감을 이끌어 나가도록 하는 것이다.

① 개인적 책무

- 학생들로 하여금 사전 과제물을 반드시 이수하도록 하고, 반드시 수업 전에 성적을 부여하도록 함
- 소규모 집단 수를 늘려서 자신의 과제물에 대한 자신의 의견을 표출할 수 있도록 함
  (e.g. 소그룹 학생 모두에게 각자 자신의 역할을 담당할 것을 요구)
- 학습평가에 동료들 간의 평가를 포함
- 수업에 대한 준비 여부(각 학생이 사전에 주어진 과제물의 학습)를 확인하는 시험 실시

학생은 자기 향상에 관한 책임을 지고 있다. 왜냐하면 이러한 수업준비 확인절차는 시험을 통해 이루어지고 그것이 궁극적으로 학습평가 일부에 포함되기 때문이다. 그룹에 대한 시험에서는 그룹에 소속한 각 개인이 자신이 선택한 답에 대한 이유와 입장을 설명하게 된다. 이러한 토론의 결과로 각 개인은 미리 준비한 과정에 대한 평가와 그 증거에 대한 즉각적인 되먹임을 받을 수 있고 그룹의 중요한 의사결정에 대해 각 개인이 자신의 의견을 표현하는 것의 중요성을 알려준다.
미리 사전 준비가 안 된 학생들은 대게 동료평가에서 낮은 평가를 받게 된다.

② **집단적 책임**

집단적 책임성이 없다면 교수나 학생들은 학습목표가 달성되었는지, 학생들이 소규모집단 수업을 진지하게 받아들이는지 확신할 수 없다. 이러한 소집단 책임수업은 소규모그룹에 대한 관리와 집단적인 전체 토론이 잘 이루어져야 한다. 성공을 위해 가장 중요한 것 중 하나는 그룹에게 주는 과제물이다. 그룹에게 주는 과제는 실제로 가시적인 결과물을 내고 학생들이 산출해 낸 결과물의 질에 대한 평가와 즉각적인 되먹임뿐만 아니라 타 그룹간의 비교가 가능한 것이어야 한다.

3 S

개인학습과 소규모토론 수업에서 과제물은 서로 연결되어있고 상호보완적이어야 한다. 이것이 잘 되었을 경우 첫 번째, 두 번째 단계에서의 과제물은 세 번째 단계에서 긍정적이고도 효과적인 배움을 가져올 수 있다. 가장 효과적인 결과를 위해서는 첫 번째 개인과 그룹에 같은 문제(same problem)를 사용해야 하고, 두 번째 개인이나 그룹은 수업의 학습 내용에 근거하여 특정답지를 선택(specific choice)해야 하고, 세 번째 동시보고(simultaneous report)이다.

즉 수업 중 토론을 통해 도출된 선택에 대한 그룹 의견을 동시에 보고하도록 한다. 이처럼 상호간에 연결되고 보완적인 과제물의 중요성은 한 교수가 의과대학생들의 비판적(진단적) 사고를 개발시키기 위해 임상사례를 사용한 경험에 잘 나타나 있다. 그 교수는 각 증례에 대해 임시적인 진단을 내리고 한 페이지의 메모로 쓰도록 하였으나 결국 수업의 결과에 대해 실망하게 되었다. 그 이유는 첫 번째로 학생들이 주어진 증례에서 일부분만을 학습하는 것이었는데 이는 학생들이 그룹 구성원 간에 학습 내용을 분배한데 그 원인이 있었다. 두 번째 이유는 학생들마다 제출한 보고서를 검사하고 교정하는데 너무 많은 시간이 걸려 제대로 피드백을 주지 못한 것이다.

결국 이러한 단점을 보완하고자 앞에서 설명한 수업준비의 확인절차를 수업 중에 갖는 것이다. 제일 먼저 개인학습에 대한 점검을 실시하여 그룹이 어떠한 결정을 이루기 전 각자가 준비해온 것에 대한 기여도를 높이게 한다. 즉 학생들이 교과내용의 기본적인 개념을 완전 숙달하게 한 후 토론에 임하게 하는 것이다. 수업 당일 증례에 대한 새로운 중요한 정보를 추가하고 각 그룹에게 일정 시간을 부여한 후 주어진 답지 중 가장 그럴듯한 진단을 고르게 하거나 아니면 주어진 정보로는 명확한 진단을 내릴 수 없는 입장임을 표명하도록 하였다. 이러한 방법을 사용하면 소그룹의 토론도 항상 활발하였고 연이은 소그룹간의 전체 토론도 매우 성공적이었다.

아이디어의 교환을 촉진시키는 학습채택

그룹토론을 통하여 학생들이 다른 학생들의 새로운 관점에 노출되기 위해서는 두 가지 요소가 매우 중요하다. 하나는 앞에서 이미 설명한 바와 같이 교수가 과제물을 통하여 소그룹 구성원들이 서로 주고받는 상호작용을 얼마나 고양시키는가 이고 다른 하나는 그룹에 존재하는 다양한 의견과 생각, 관점이다.

① 그룹상호작용을 요구하는 과제물의 사용

학생들의 토론이 활발해지지 못하는 가장 큰 이유는 과제물이 너무 쉽거나 또는 한 학생이 나머지 그룹 모두를 위하여 일을 하는 것이다. 반면에 과제물로 너무나 방대한 양의 보고서를 요구하는 것은 학생들의 학습이나 상호작용을 제한하게 된다. 만약 아주 긴 보고서를 요구할 경우 그룹 토론은 별로 효과적이지 않다. 그 이유는 보고서 작성에 대한 학생들의 압박감으로 실제 토론시간이 짧아지고 과제를 분배함으로써 과제 전체를 보지 못하게 되기 때문이다.

반면에 학생들로 하여금 교과내용의 개념을 이용하여 어려운 선택을 하게 하면 소그룹 내 상호작용과 학습을 극대화시킨다.

② 참여에의 장애물 제거

간혹 새로 그룹을 구성하게 되면 일부 학생은 이야기하기를 꺼려한다. 이럴 때는 사회자나 서기와 같이 그룹 내에 역할을 분담시키는 것이 해결 방법이 될 수 있다. 가장 좋은 것은 동일한 그룹을 일정 기간 지속시키는 것이며 집단의 결속력을 키울 수 있는 과제물이나 평가 방법을 사용하는 것이다.

그룹이 형성되고 점차 그룹에 대한 결속력이 강해지면 구성원들 간에 신뢰가 형성되고 오해나 감정 훼손에 대한 우려가 점차 사라지게 되며 말이 없던 구성원들이 점차 토론에 참여하게 된다.

그리고 구성원 개인의 성공이 그룹의 성공에 연결되어 있음을 경험하게 되면서 구성원들은 더욱 동기가 부여되고 타 구성원에 대한 배려를 하게 된다.

③ 수업시간의 그룹 활동

학생들의 효율적인 상호작용을 위해서는 학생들이 수업시간에 집단 활동을 갖도록 허용해야 한다. 대개의 조 활동은 수업시간 외에 학생들끼리 만나서 작업을 하도록 하는데, 학생들은 이것이 번거롭고 귀찮아서 주어진 과제물을 분배한 다음 서로 조합해서 결과물을 제출하게 된다. 이렇게 되면 그룹 구성원 간에 배우는 것은 매우 좁아지고 그룹의 결과물은 이름일 뿐이게 된다.

④ 다양한 그룹의 형성

그룹은 최소한 5-7명이 되어야 하고 다양할수록 좋다. 학생들을 무작위로 배치할 수 있으면 좋다. 남녀의 적절한 비율은 반드시 고려해야 할 사항 중 하나이다. 그리고 전문대학원 학생일 경우 입학 전의 전공이라든가 직장경력 등도 고려해 볼만하다. 그러나 중요한 것은 모든 그룹에 비슷한 조건을 만들어 주는 것이다.

Team-Based Learning – TBL (Michaelsen, Knight & Fink, 2002)

Team-based learning 작성 양식

1. 학습 목표에 따른 주제를 정한다.

2. 주어진 시간에 다룰 양을 정한다.

3. 사전에 과제물을 주고 학습을 하고 오도록 한다.

4. Readiness Test 를 준비한다.

5. 응용과제를 준비한다.

6. 평가를 어떻게 할 것인지 정한다.

7. 학생에게 동기 부여 방법을 고안한다.

문제바탕학습(Problem Based Learning)

PHASE 1: Individual Readiness Assurance Test

Defining Problem-Based Learning

1. PBL 수업을 할 때 예상하는 것은?
   1) 기초의학 교육을 충분히 하기 어렵다.
   2) 같은 내용을 배우는데 시간이 더 오래 걸려 효율이 떨어진다.
   3) 증상과 관련된 다양한 면을 학습하느라 지식의 암기가 어렵다.
   4) 문제를 해결하는데 노력하느라 지식을 습득하는 정도가 미흡하다.
   5) 새로운 문제에 도전하는 학생들의 마음가짐이 없이 성공을 장담할 수 없다.

2. PBL 수업의 특징은?
   1) PBL은 교육자 중심이다.
   2) PBL은 자기주도 학습능력이 강조된다.
   3) PBL에서 교사는 '지식 전달자'의 역할을 한다.
   4) PBL은 협동학습 보다는 개인학습을 강조한다.
   5) PBL에 사용되는 문제는 하나의 정확한 답이 구해지는 상황을 사용한다.

3. Barrows와 Tamblyn에 의하면 PBL은 어떻게 정의되는가?
   1) 학생들이 tutor의 관리 하에 소집단으로 문제를 다루는 학습
   2) 문제에 대한 이해나 해결책을 향한 활동으로 인해 초래된 학습
   3) 실생활의 문제에 대해 비판적 사고, 문제해결력을 적용하는 교수방법
   4) 문제해결력과 내용을 가르치고 자기 주도적 학습을 위해 설계된 교수전략
   5) 기초 및 임상의학지식을 습득하는 방법으로 환자의 문제를 활용하는 교수방법

4. PBL 수업의 진행과정으로 올바른 순서는?
   ① 알아야 사항(action plan) 정리
   ② 개별학습
   ③ 자료(data)의 수집 및 문제(problem) 분류
   ④ 소집단학습
   ⑤ 가설 설정
   ⑥ 학습할 내용(learning issues) 정리

   1) ④-①-③-⑤-②-⑥
   2) ①-⑥-②-⑤-③-④
   3) ③-①-⑤-④-⑥-②
   4) ③-⑤-②-⑥-①-④
   5) ③-⑤-①-⑥-②-④

문제바탕학습(Problem-based learning)

21세기 정보화, 무한 경쟁사회에 능동적으로 대처할 수 있는 사람을 기르기 위해서는 무엇보다 창의력, 비판적 사고력, 유연한 문제해결력, 정보를 효율적으로 분석하고 종합할 수 있는 능력 등이 요구된다. 강의위주의 암기식, 강사중심, 교과서 중심의 수업방식으로는 21세기 사회가 필요로 하는 창의력이나 문제해결력을 비롯한 고등정신능력을 함양하는 데에 어려움이 있다는 것을 인식하고, 그 대안으로 제시된 새로운 학습전략 중의 하나가 문제바탕학습(Problem-Based Learning: PBL)이다.

문제바탕학습은 1960년대 중반 Barrows에 의해 캐나다의 McMaster 의과대학에서 처음 실시되었다. 여기에서 학습이란 학습자의 인지구조의 계속적인 변화로 단순한 양적 변화뿐만 아니라 질적 변화도 포함시키며 경우에 따라서는 문제의 답을 가르치기보다는 문제를 해결하는 방법을 가르치는 것이 더욱 강조된다.

의과대학에서 문제바탕학습은 실제 환자를 만나는 것처럼 체계적으로 만들어진 가상의 시나리오를 학생들에게 단계적으로 주어서 학생들이 소집단 토론을 통해 환자를 진단하고 치료 계획을 세우는 과정을 경험하게하고 그에 필요한 지식을 찾아내어 스스로 공부하게 하는 일종의 교육방법이다. 학생들은 질병이 아니라 환자의 문제를 중심으로 학습하고 자신의 지식과 경험을 중심으로 문제를 해결하며, 토론과 성찰과정을 거치면서 개인 경험의 객관화를 통해 지식의 전이를 경험한다.

Barrows와 Tamblyn(1980)은 문제바탕학습을 ‘문제에 대한 이해나 해결책을 향한 활동의 과정으로 초래된 학습’으로 정의한다. 학습자들은 학습과정의 처음부터 문제에 직면하고, 이 때 제시되는 문제는 문제해결력이나 추론능력의 적용과 문제해결에 필요한 지식 습득을 위한 자극으로서의 역할을 한다.

학생들은 문제바탕학습을 통해서 의학지식을 구조화하고 조직화해서 임상에 접했을 때에 유용하게 활용할 수 있는 능력을 갖추게 된다. 학생자신이 자기의 인식과정과 그 과정에서 나타난 결과를 총괄적으로 고찰할 수 있는 능력을 메타인지(meta cognition)라고 하는데 문제바탕학습에서는 메타인지와 같은 고등정신능력이 필수적이다.

문제바탕학습의 특징 : Barrows(1996)에 따르면, 문제바탕학습은 첫째, 학습자를 중심으로 둘째, 소집단 내에서 일어나며 셋째, 튜터는 촉진자 또는 안내자의 역할을 하고 넷째, 학습의 자극제로 문제가 사용되며 다섯째, 자기주도 학습이 강조된다는 특징을 가진다.

(1) 문제바탕학습은 학습자 중심이다. 학습자는 문제해결자로 학습에 참여하게 되는데, 좋은 해결책을 위해 필요한 조건과 근본 문제를 확인하며 필요한 많은 정보들을 직접 다루면서 의미와 이해를 추구하고 학습에 대한 상당한 책임을 맡는다. 학습자 자신의 학습이 ‘얼마나’, ‘어떻게’ 이루어 졌는지 그리고 ‘무엇이’ 앞으로 더욱 필요한지를 스스로 인식하고 깨달아야 하는데, 이는 단지 학습의 효과 측면에서뿐만 아니라 스스로에게 학습의 내재적 동기부여라는 측면에서도 중요하다.

(2) 문제바탕학습은 협동학습을 강조한다. 학습활동은 크게 집단학습과 개별학습으로 구분되며 문제해결과정은 문제제시, 자기주도학습, 협동학습, 일반화, 성찰 등의 단계를 거친다. 문제바탕학습에서는 특히 소그룹 활동을 통한 협동학습이 강조된다. 먼저, 학습자들이 문제를 받으면 다 같이 모여서 문제에

대한 해결안과 관련된 생각을 다시 세분화하여 개개 학습자에게 자율학습시간에 다루게 될 과제를 부여하게 된다. 이어서 학습자들은 개인적으로 자율적 학습과정을 통해서 부여된 과제를 완수하며 다시 모두 모여 자신의 생각을 정리 한다. 이후 협동학습과정을 통해 제시되었던 문제해결과 관련된 많은 생각이 수정되고 결국, 처음과 비교해 볼 때 질적으로 상당히 발전된 결과를 얻는다.

(3) 문제바탕학습은 교사의 역할을 '지식 전달자'에서 '학습 진행자'로 전환시킨다. 튜터는 안내자, 인도자, 촉진자, 상담자, 조력자, 관리자 등 여러 가지 이름으로 불리며 소집단학습과 자율학습이 원활하게 이루어지도록 촉진하는 사람이다. 튜터가 없는 집단에서 학습한 학생들은 튜터가 있는 집단에서 학습한 학생들보다 학습목표를 정의하는데 더 어려움을 겪었다는 연구가 있는데 이는 문제바탕학습이 아무리 학생중심의 학습을 표방하더라도 튜터의 역할이 학생들의 학습이 성공적으로 이루어지기 위한 필수요인이라는 것을 보여준다.

(4) 문제바탕학습은 '문제'로 시작된다. 문제는 너무 쉽게 해결되거나 특정한 틀에 얽매여 하나의 정확한 답이 구해지는 상황이 아니라 비구조화 되고 설명을 필요로 하는 일련의 현상군을 의미한다. 의과대학의 문제바탕학습에서 문제란 환자의 증상 등 의학적인 문제뿐만 아니라 환자가 처한 심리적, 가족적, 사회적, 경제적 문제 등을 포괄하는 것으로, 좋은 문제란 임상에서 흔히 접할 수 있는 문제 상황이나 흔하지는 않지만 진단을 놓치면 심각한 결과를 초래하는 문제 상황 또는 에이즈와 같이 사회, 경제적으로 심각한 영향을 미치는 문제 상황, 기초의학 분야에서 중요한 개념을 심어줄 수 있는 문제 상황 등을 의미한다.

(5) 문제바탕학습은 자기주도 학습능력이 강조된다. 학습자에게 경험 가능한 실제적 문제 상황 제시와 함께 해결자의 역할을 부여함으로써 학습자로 하여금 학습에 대한 주인의식을 갖고 문제를 해결하도록 장려한다. 학습자 자신이 무엇을 알아야 하고 무엇을 알고 싶은지를 발견할 수 있다는 사실을 자각하게 하고, 분명한 해답이 없는 상황에서 해결책을 만들기 위해 새로운 정보를 사용할 줄 안다는 사실도 깨닫게 한다.

문제바탕학습의 장점 : 이 방법은 지식의 습득뿐 아니라 환자의 문제를 해결하는 방법을 깨우치는 데에도 도움이 된다. 학생들은 자신이 공부한 지식, 개념, 수기 등을 환자의 증례와 관련지어 기억한다. 이렇게 함으로써 관련된 또 다른 문제를 만났을 때 쉽게 기억을 해 낼 수 있으며 기억이 더 확고해질 수 있다.

애매한 문제를 공부하면서 학생들은 문제를 해결하고 진단하는 임상적 추론 관정을 익힌다. 또, 정보를 찾아서 분석하고 통합하고 가설을 세우며 교육적인 추론 과정을 익힌다. 이러한 과정은 학생들에게 동기부여가 되고 앞으로 경험하게 될 상황들에 대해 미리 도전해 보게 한다.

많은 연구에서 의사의 진단이나 임상적 추론의 효율성은 이전 환자들로부터 경험하고 학습한 내용과 관련이 있었다. 문제바탕학습으로 공부를 하려면 학생들은 많은 건강 문제 학습에 적극적으로 참여해야 한다.

환자의 문제를 중심으로 공부하면 학생들은 공부한 것들이 어떻게 서로 연관되는지 알게 되고 특히 기초의학의 중요성을 알게 된다. 또, 앞으로 전문가로서 꾸준히 환자 문제를 학습하는데 필요한 수기들을 익힐 수도 있다.

이러한 방법에 잘 접근할 수 있는 교사들은 문제바탕/학생중심의 교육의 장점을 발견하게 되는데 이러한 방법이 재미있고 가치 있으며 보다 자연스럽고 시간이 더 절약된다는 점이다. 학생중심의 작업을 통해 학생들은 교사들의 역할을 일부 수행하면서 즐거워하고 동기부여를 받으며 스스로 어른이 된 느낌을 받고 임상적 추론이나 학습능력이 향상되며, 기초 지식을 얻는다.

문제바탕학습의 단점 : 문제바탕/학생중심의 학습은 확실하지 않고 일종의 퍼즐 같은 문제를 다루면서 문제해결력을 키우고 자기주도 학습을 하는 학생들의 마음가짐이 중요하다. 교사는 학생들이 이러한 과정을 잘 수행하도록 안내할 수 있는 능력이 필요하고 문제바탕학습 자료를 만들고 모아서 제공하는 것이 필요하다.

간혹 문제바탕학습의 문제점으로 대두되는 것들이 있는데 첫째, 이 방법이 환자를 평가하고 치료하는데 중점을 두어서 기초의학에 대한 학습이 소홀해 지지 않을까 하는 것이고 둘째, 문제해결능력에 중심을 두어 지식 습득이 미흡할까 하는 것이다. 그러나 문제바탕학습이 잘 수행된다면 두 가지 모두 나타나지 않는다. 교사로부터 충분히 안내를 받으면 기초와 임상의학의 다양한 부분들을 학습할 수 있는데 환자의 문제를 단순히 진단하고 감별진단 하는 것에 멈추지 않고 해부학, 생화학, 생리학적 기전도 학습하게 할 수 있다. 가장 중요한 점은 그 문제가 갖는 교육목표를 충분히 이해하는 것이다. 이는 학생과 교사에게 사용되는 문제를 통해 어떤 내용을 다루어야 할 지 지침을 통해 안내하는 것이 필요하다.

셋째로 문제바탕학습이 비효율적인 학습이라 생각할 수 있다. 낯선 문제를 만나면 학생들은 용어를 이해하고 증상이나 징후의 의미를 파악하고 관련된 해부학이나 생리학적 지식이나 사회적, 역학적, 심리적인 면까지를 이해하느라 많은 시간이 필요하다. 모든 문제들은 관련된 많은 중요한 영역들을 공부해야 하므로 새로운 영역의 첫 번째 문제를 다룰 때에는 시간이 많이 필요하다. 그러나 이러한 작업이 다른 문제를 해결하는데 기본 바탕이 되므로 사실 그다지 효율이 떨어지는 것은 아니다. 학생이 기본 지식을 갖춘다면 이 첫 번째 문제에서 얻은 지식을 바탕으로 이어지는 문제들에 쉽게 접근할 수 있고 이 과정을 통해 학습된 지식들이 서로 연결되고 강화될 것이다.

문제바탕학습에서의 평가 : 이 방법으로 학습한 학생들은 문제를 제시했을 때 기억력이 가장 잘 살아난다. 문제바탕학습에서는 학생들이 문제를 잘 해결하는지, 또 학습한 내용을 잘 이용하는지와 같은 내용을 평가하는 것이 필요하다. 학습을 개선할 목적으로 학습과정 중에 실시하는 평가를 과정지향평가(progress oriented assessment)라 하며 이를 통해서 학생들은 평점을 받을 뿐 아니라 되먹임을 통해 앞으로의 학습을 개선하게 된다. 여기에는 튜터평가, 자기평가, 동료평가, 우회측정, 삼단계시험 등이 포함된다.

학습목표에 대한 성취도를 최종적으로 판정하기 위한 평가를 결과지향평가(outcome oriented assessment)라 하며 개념도, 선다형 객관식 시험, 변형논술문제, 컴퓨터 임상모의 시험, 객관구조화진료시험(objective structured clinical examination: OSCE), 점진적 검사 등이 포함된다.

기생충학 수업 계획서(2025년도 제 2학기) - 제 32판

Lecture and Laboratory Schedule for Parasitology (2025)
Department of Parasitology, Hallym University, 32nd ed.

Editor: Sun Huh
Publisher: Department of Parasitology, Hallym University, Chuncheon 24252, Korea
Tel: 033.248.2652

* Not for sale

The first edition received the Korean Association University Education Award for the Best Syllabus for Class, In April 1, 1994.

Copyright (C) 2025. 10. 27. Department of Parasitology, Hallym University
Creative Commons license: CC-BY
