# Supplementary figures and images for: Implementation of artificial intelligence in the 2025 medical parasitology course at Hallym University
Source: J Educ Eval Health Prof. 2026 Feb 5;23:4. doi: 10.3352/jeehp.2026.23.4 (PMC12976625; doi:10.3352/jeehp.2026.23.4)

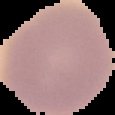

Supplement: Supplementary file 2 — Supplement 2. The image dataset used for deep learning training on malaria parasite detection was selected from the dataset used by Reddy et al. [4]. [file jeehp-23-04-suppl2.zip › test_images/test_ uninfected.png]

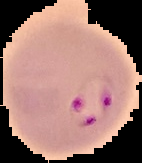

Supplement: Supplementary file 2 — Supplement 2. The image dataset used for deep learning training on malaria parasite detection was selected from the dataset used by Reddy et al. [4]. [file jeehp-23-04-suppl2.zip › test_images/test_parasitized.png]
