# Supplementary material for: Implementation of artificial intelligence in the 2025 medical parasitology course at Hallym University
Source: J Educ Eval Health Prof. 2026 Feb 5;23:4. doi: 10.3352/jeehp.2026.23.4 (PMC12976625; doi:10.3352/jeehp.2026.23.4)
Supplement: Supplementary file 3 — Supplement 3. The author’s answer to the AIMP final exam prompt on malaria parasite detection. [file jeehp-23-04-suppl3.pdf]

하은희 기생충 AI 문제 답

2025.12.15

각 조별로 아래 두 문항을 해결하고 각자 최소 하나의 문항을 해결한 답을 각자 notion 에올리시오.

선택 문항: 문항 1번

- Malaria 감염 RBC와 비감염 RBC사진을 Malaria Cell Images Dataset https://www.kaggle.com/datasets/iarunava/cell-images-for-detecting-malaria/data에서 내려 받아, 이미지 분류 CNN을 설계해 감염비감염 적혈구를 구분하는 모델을 만들고 모델의 성능을 평가한 보고서를 작성하시어 각자 노션에 올리시오.

  참고문헌>https://pmc.ncbi.nlm.nih.gov/articles/PMC12629934/#S2

본 보고서에서 2가지 버전 ((1) Teachable Machine 그리고 (2) Vibe Coding)을 이용하여 선택 문항 1번 모델을 만들었습니다.

(1) Teachable Machine을 활용한 이미지 분류

Image Model - Teachable Machines 를 사용하여 이미지 분류를 실행했습니다.

고급 설정

모델이 얼마나 잘 작동하는지 파악하는 데 도움이 되는 몇 가지 그래프입니다.

처음에는 이해가 되지 않아도 걱정하지 마세요. Teachable Machine을 사용하는 데는 이 그래프가 필요하지 않습니다. 사실 대부분의 사람들은 그래프를 굳이 사용하지 않습니다. :)

어휘

클래스별 정확도

CLASS	ACCURACY	# SAMPLES
Parasitized	0.87	15
Uninfected	1.00	15

혼동 행렬

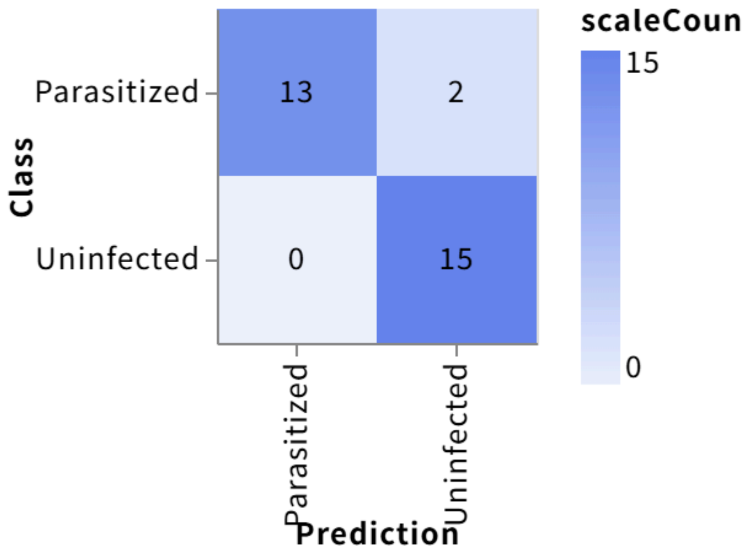

에포크별 정확도

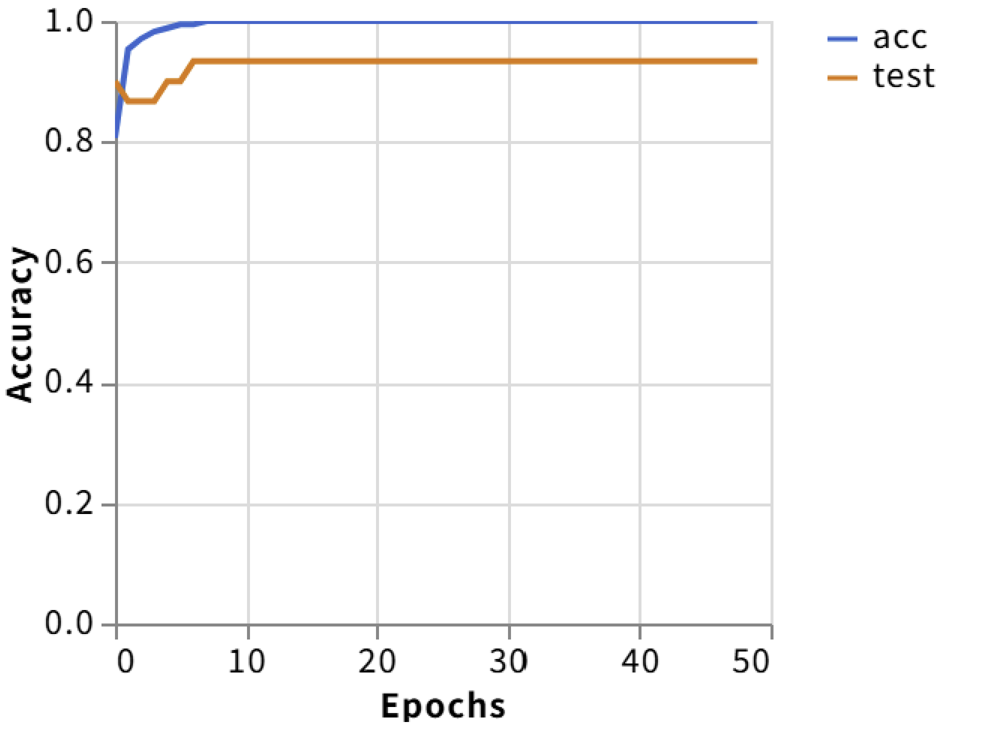

에포크별 손실

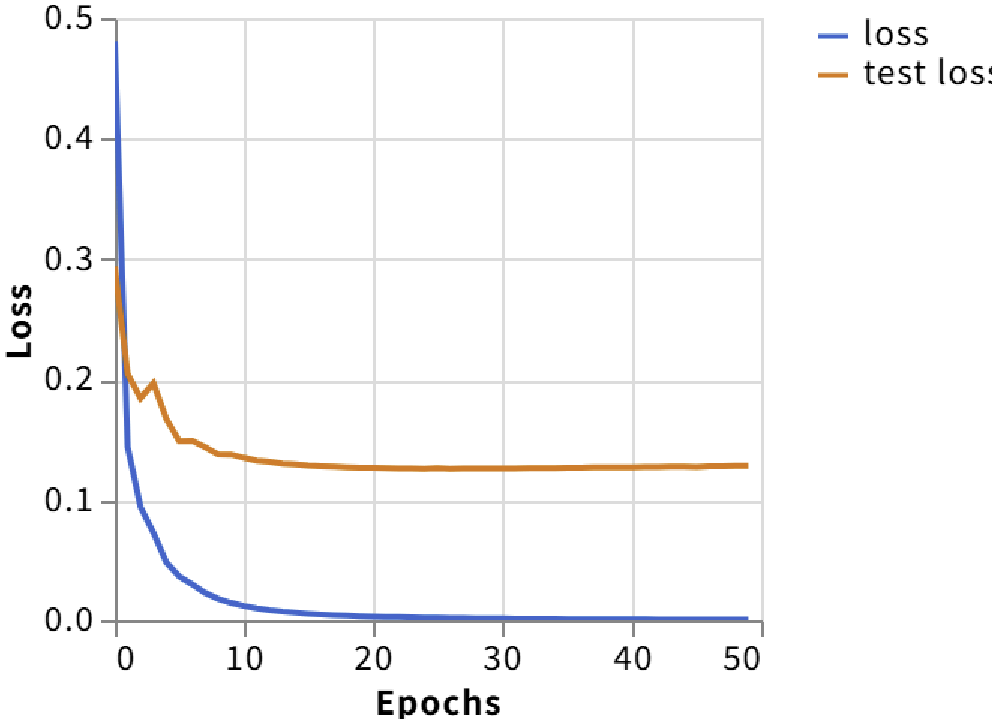

Test (Parasitized Test Image)

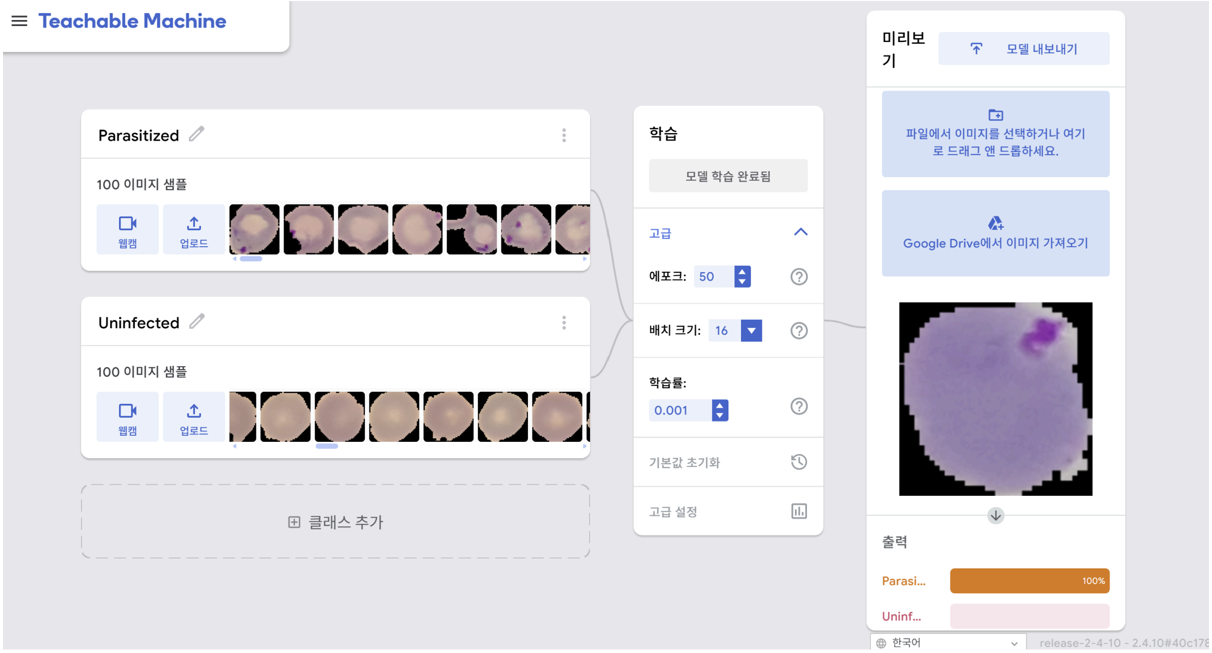

Test (Uninfected Test Image)

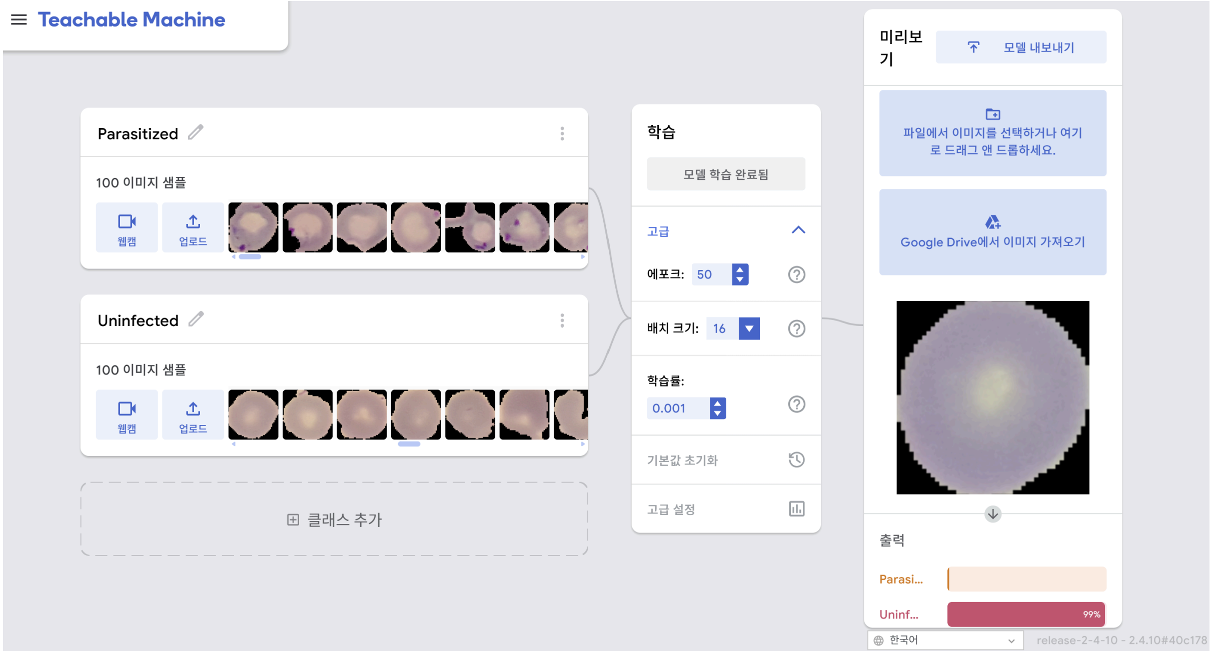

AI Colab Result (모델의 성능을 평가한 보고서)

https://colab.research.google.com/drive/1QgIDN1QipXbNsAywlqb2v2-aadjnvIlz?usp=sharing

사용한 Test Image (test_images.zip)와 모델 (converted_tflite.zip)

test_images.zip

converted_tflite.zip

(2) Vide Coding 을 이용한 분류

aistudio.google.com 를 활용하여 Parasite와 uninfected 를 분류하는 모델을 만들었습니다.

User Prompt: “**https://www.kaggle.com/datasets/iarunava/cell-images-for-detecting-malaria/data** 이미지 분류 CNN을 설계해 감염비감염 적혈구를 구분하는 모델을 만들고 모델의 성능을 평가한 보고서를 작성해줘.”

생성모델:

https://ai.studio/apps/drive/1S4y1SMiwQXDSmqUQfpm1w-sLDA7FvuEY?fullscreenApplet=true

https://aistudio.google.com/app/prompts?state={"ids":["1S4y1SMiwQXDSmqUQfpm1w-sLDA7FvuEY"],"action":"open","userId":"117329364207480471566","resourceKeys":{}}&usp=sharing

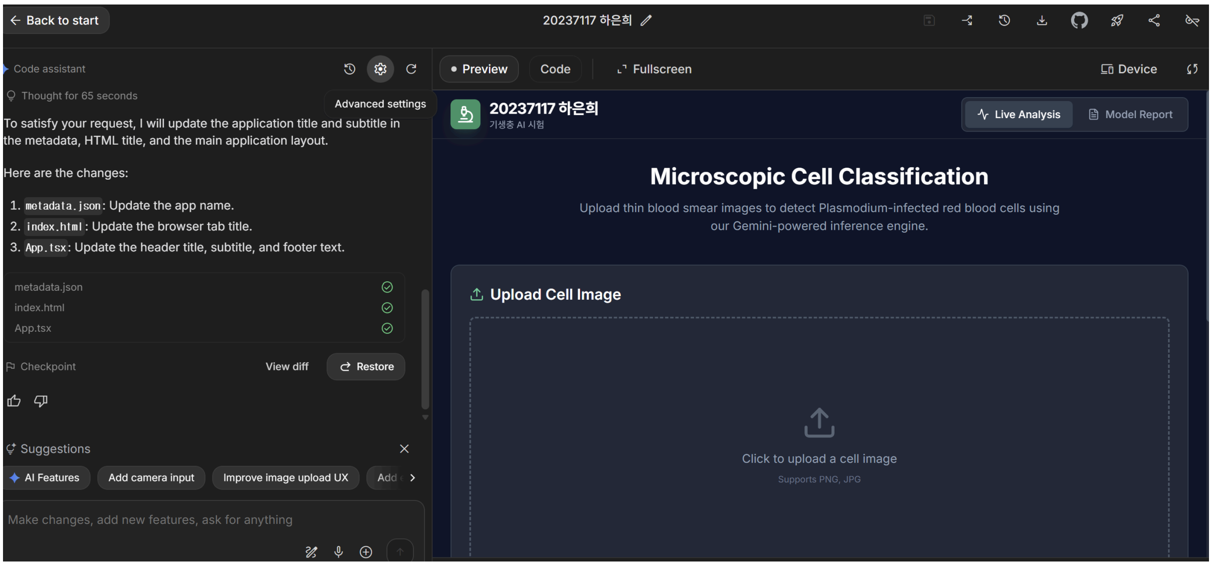

Test (Parasite Sample)

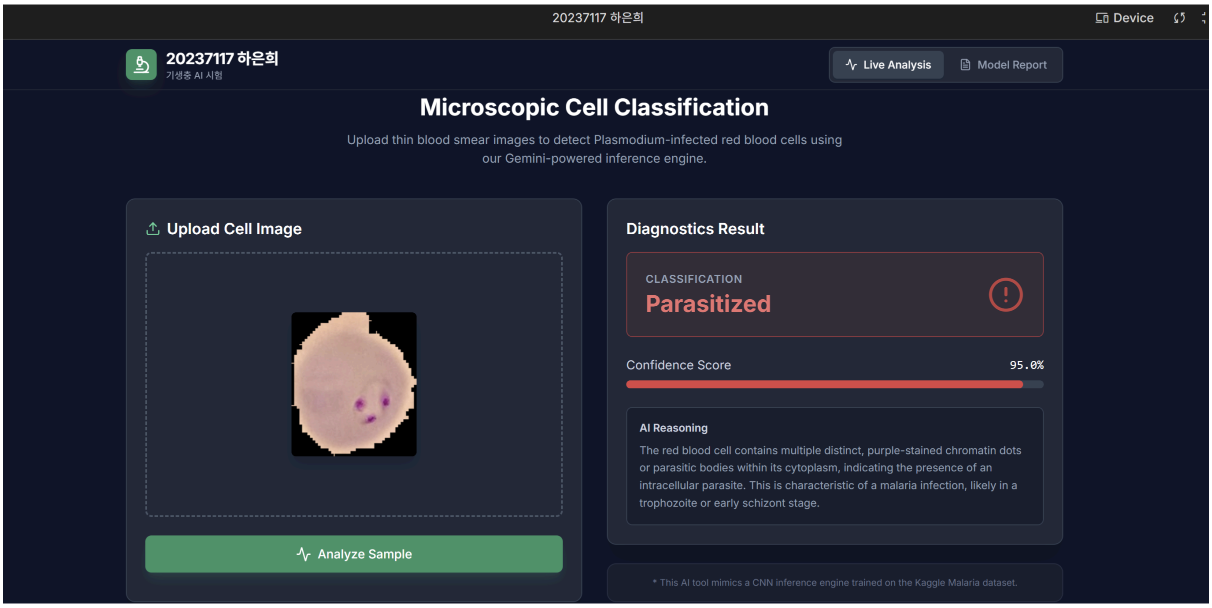

Test (Uninfected Sample)

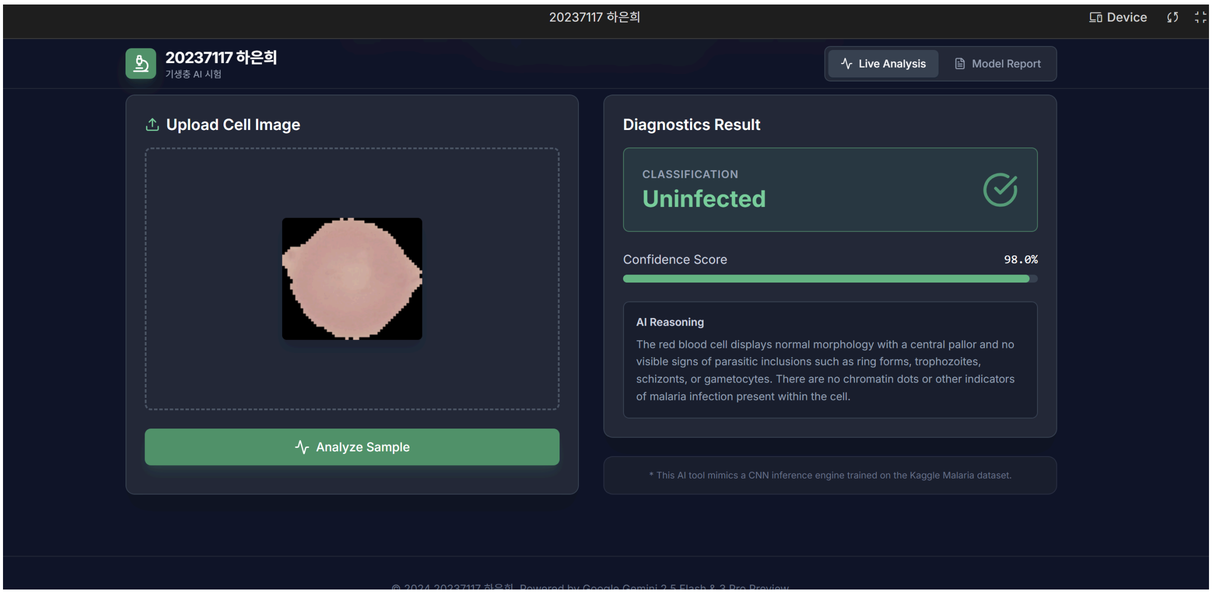

AI Studio Code:

```
import React, { useState } from 'react';
import { AppMode } from './types';
import { ImageAnalyzer } from './components/ImageAnalyzer';
import { ReportGenerator } from './components/ReportGenerator';
import { Microscope, FileText, Activity } from 'lucide-react';

const App: React.FC = () => {
  const [mode, setMode] = useState<AppMode>(AppMode.ANALYSIS);

  return (
    <div className="min-h-screen bg-slate-900 text-slate-200 selection:bg-emerald-500/30 pb-12">
      {/* Header */}
      <header className="bg-slate-900 border-b border-slate-800 sticky top-0 z-50 backdrop-blur-md bg-opacity-80">
        <div className="max-w-7xl mx-auto px-4 sm:px-6 lg:px-8 h-16 flex items-center justify-between">
          <div className="flex items-center gap-3">
            <div className="w-10 h-10 bg-emerald-600 rounded-lg flex items-center justify-center shadow-lg shadow-emerald-600/20">
              <Microscope className="text-white w-6 h-6" />
```

```
            </div>
            <div>
              <h1 className="text-xl font-bold text-white trac
king-tight">20237117 하은희</h1>
              <p className="text-xs text-slate-400">기생충 AI
시험</p>
            </div>
          </div>

          <nav className="flex items-center gap-1 bg-slate-800
p-1 rounded-lg border border-slate-700">
            <button
              onClick={() => setMode(AppMode.ANALYSIS)}
              className={`px-4 py-2 rounded-md text-sm font-me
dium transition-all flex items-center gap-2 ${
                mode === AppMode.ANALYSIS
                  ? 'bg-slate-700 text-white shadow-sm'
                  : 'text-slate-400 hover:text-slate-200'
              }`}
            >
              <Activity className="w-4 h-4" />
              Live Analysis
            </button>
            <button
              onClick={() => setMode(AppMode.REPORT)}
              className={`px-4 py-2 rounded-md text-sm font-me
dium transition-all flex items-center gap-2 ${
                mode === AppMode.REPORT
                  ? 'bg-slate-700 text-white shadow-sm'
                  : 'text-slate-400 hover:text-slate-200'
              }`}
            >
              <FileText className="w-4 h-4" />
              Model Report
            </button>
          </nav>
        </div>
      </header>

      {/* Main Content */}
```

```
      <main className="max-w-7xl mx-auto px-4 sm:px-6 lg:px-8
py-8 animate-fade-in">
        {mode === AppMode.ANALYSIS ? (
          <div className="space-y-6">
            <div className="text-center max-w-2xl mx-auto mb-1
0">
              <h2 className="text-3xl font-bold text-white mb-
3">Microscopic Cell Classification</h2>
              <p className="text-slate-400">
                Upload thin blood smear images to detect Plasm
odium-infected red blood cells using our Gemini-powered infere
nce engine.
              </p>
            </div>
            <ImageAnalyzer />
          </div>
        ) : (
          <div className="space-y-6">
             <div className="flex items-center justify-between
mb-8">
               <div>
                  <h2 className="text-3xl font-bold text-white
mb-2">CNN Architecture & Performance</h2>
                  <p className="text-slate-400">Design, train
(simulated), and evaluate custom CNN models on the Malaria Cel
l Images dataset.</p>
               </div>
               <div className="hidden lg:block text-right">
                 <p className="text-xs text-slate-500 uppercas
e tracking-wider">Dataset Source</p>
                 <a href="https://www.kaggle.com/datasets/iaru
nava/cell-images-for-detecting-malaria" target="_blank" rel="n
oreferrer" className="text-blue-400 hover:text-blue-300 text-s
m font-medium">Kaggle Malaria Dataset ↗</a>
               </div>
             </div>
            <ReportGenerator />
          </div>
        )}
      </main>
```

```
      {/* Footer */}
      <footer className="border-t border-slate-800 mt-12 py-8
text-center text-slate-500 text-sm">
        <p>© 2024 20237117 하은희. Powered by Google Gemini 2.5
Flash & 3 Pro Preview.</p>
      </footer>
    </div>
  );
};

export default App;
```
